# ID 48 - Eficácia e Segurança Comparada de Formulações Intravenosas de Ferro para Tratamento de Anemia: revisão sistemática e metanálise em rede

**DOI:** 10.5327/2237-9622.2025.v34s1.48

**Published:** 2025-11-25

**Authors:** Francino Machado de Azevedo, Layssa Andrade Oliveira, Haliton Alves Oliveira, Rosa Camila Lucchetta

**Keywords:** anemia ferropriva, ferro, revisão sistemática, metanálise em rede

## Abstract

**Introdução:**

A anemia por deficiência de ferro (ADF) é a maior causa de morbidade em todo o mundo, afetando mais de 1,9 bilhão de pessoas. Tradicionalmente, a abordagem da maioria dos casos de ADF é feita com reposição oral de ferro, todavia, nos últimos anos o uso do ferro intravenoso (IV) como primeira opção de tratamento tem ganhado a preferência de clínicos. Entretanto, há escassez de evidências sobre eficácia comparativa das opções para reposição de ferro IV. Nesse sentido, o objetivo deste estudo foi avaliar a eficácia e a segurança das soluções intravenosas de ferro para o tratamento da ADF.

**Método:**

Trata-se de uma revisão sistemática com metanálise em rede (NMA). Foram recuperados estudos das bases PubMed, Embase e Cochrane library. Foram incluídos ensaios clínicos randomizados (ECR) que tivessem como intervenção qualquer tipo solução IV de ferro e que abordassem desfechos de eficácia (alteração na hemoglobina [Hb]) e segurança (eventos adversos sérios [EAS]). Foi avaliado o risco de viés por meio da ferramenta ROB2. Uma metanálise em rede frequentista foi realizada, sendo reportada as medidas de diferença média (DM), risco relativo (RR) como medida de efeito. Pressupostos de transitividade e consistência foram testados para validar a NMA. A certeza da evidência foi avaliada por meio do CINeMA.

**Resultados:**

Foram incluídos 34 ECR nesta revisão sistemática. Carboximaltose férrica (58,8%) foi a intervenção mais presente nos ECR, de outro modo, sacarato de hidróxido férrico e gluconato férrico de sódio foram avaliados em apenas 2,9% dos estudos incluídos. Foi possível realizar NMA para dois grupos de participantes: com doença renal crônica (DRC) e de etiologias não especificadas (ENE). Para DRC, as estimativas mistas da NMA mostram que carboximaltose, ferumoxytol e derisomaltose podem ser as terapias que mais elevam Hb em duas semanas comparado à sucrose férrica. Entre pacientes com ENE, carboximaltose (DM=0,98 IC 95% 0,47-1.48)) e derisomaltose (DM=0,69 IC 95% 0,29-1,08), podem ser as alternativas mais eficazes. Em relação à segurança, observou-se que a derisomaltose como a tecnologia com menor incidência de EAS (DRC RR 0,76 IC 95% 0,46-1,27; ENE RR 0,83 IC 95% 0,20-3,49) comparado à sucrose férrica), posicionando-se com a mais segura em ambos os grupos de pacientes.

**Conclusão:**

Essa é a primeira NMA que compara todas as formulações IV de ferro disponíveis em diferentes subgrupos de pacientes. Os achados são consistentes a estudos prévios que demonstram eficácia destacada da carboximaltose e derisomaltose. Todavia, a presença do ferumoxytol como alternativa eficaz chama atenção, visto que a tecnologia ainda não dispõe de registro no Brasil. Em outra direção, os resultados de segurança parecem clarear as dúvidas existente sobre a carboximaltose, que embora eficaz, apresenta padrão de segurança inferior aos das tecnologias de eficácia semelhante.

